# The triglyceride-glucose index is a predictor for cardiovascular and all-cause mortality in CVD patients with diabetes or pre-diabetes: evidence from NHANES 2001–2018

**DOI:** 10.1186/s12933-023-02030-z

**Published:** 2023-10-17

**Authors:** Qin Zhang, Shucai Xiao, Xiaojuan Jiao, Yunfeng Shen

**Affiliations:** 1 Department of Metabolism and Endocrinology, The Second Affiliated Hospital of Nanchang University, Jiangxi, Nanchang, 330006 China; 2 Department of Cardiovascular Medicine, The Second Affiliated Hospital of Nanchang University, Jiangxi, Nanchang, 330006 China

**Keywords:** Triglyceride-glucose index, Mortality, Cardiovascular Disease, NHANES

## Abstract

**Background:**

The association between the triglyceride-glucose (TyG) index and mortality in cardiovascular disease (CVD) patients with diabetes or pre-diabetes remains unclear. This study aimed to investigate the relationship between baseline TyG index and all-cause and cardiovascular (CV) mortality in CVD patients with diabetes or pre-diabetes among American adults. .

**Methods:**

This study enrolled 1072 CVD patients with diabetes or pre-diabetes from the National Health and Nutrition Examination Survey (2001–2018). Mortality outcomes were determined by linking to National Death Index (NDI) records up to December 31, 2019. Multivariate Cox proportional hazards models were constructed to analyze explore the associations between baseline TyG index and mortality. Non-linear correlations were explored using restricted cubic splines, and a two-piecewise Cox proportional hazards model for both sides of the inflection point was constructed.

**Results:**

During 7541 person-years of follow-up, a total of 461 all-cause deaths and 154 CVD-related deaths were recorded. The restricted cubic splines revealed a U-shaped association between the baseline TyG index with all-cause and CVD mortality in CVD patients with diabetes or pre-diabetes. Specifically, baseline TyG index lower than the threshold values (TyG index < 9.05 in all-cause mortality and < 8.84 in CVD mortality) was negatively associated with mortality (HR 0.47, 95% CI = 0.27–0.81 for all-cause mortality and HR 0.25, 95% CI = 0.07–0.89 for CVD mortality). In contrast, baseline TyG index higher than the threshold values (TyG index > 9.05 in all-cause mortality and > 8.84 in CVD mortality) was positively associated with mortality (HR 1.42, 95% CI = 1.02–1.99 for all-cause mortality and HR 1.77, 95% CI = 1.08–2.91 for CVD mortality).

**Conclusions:**

A U-shaped association was observed between the baseline TyG index with CVD and all-cause mortality in CVD patients with diabetes or pre-diabetes in a American population. The thresholds of 8.84 and 9.05 for CVD and all-cause mortality, respectively.

## Background

With the accelerated aging process, the morbidity and mortality of cardiovascular disease (CVD) continue to rise, and the high incidence of CVD is becoming an important public health problem [1]. In 2019, the number of CVD patients in 204 countries and regions increased from 271 million in 1990 to 252 million, and the number of CVD deaths increased by 6.5 million, making it a leading cause of mortality worldwide [2]. Despite significant progress in treating major heart and circulatory disorders over the past two decades, the number of deaths from CVD remains the highest, posing a serious threat to human life [1]. It is worth noting that type 2 diabetes mellitus (T2DM) and pre-diabetes are prevalent in patients with established CVD, and are associated with poor outcomes [3]. Identifying residual risk factors of CVD patients with different glucose metabolism statuses is critical for reducing mortality, particularly the risk of cardiovascular death. Insulin resistance (IR) is a state of decreased sensitivity and responsiveness to the action of insulin, often occurring several years before the onset of diabetes [4]. There has been increasing evidence indicating that IR and associated disorders contribute to the development of CVD in diabetic as well as nondiabetic subjects [5]. It is well known that individuals with IR have a higher likelihood of developing metabolic disorders like hyperglycemia, dyslipidemia, and hypertension, all of which are strongly associated with unfavorable outcomes in cardiovascular disease. Thus, IR is considered both a pathogenic factor and an indicator of poor prognosis in individuals with CVD, whether they have diabetes or not. The triglyceride-glucose (TyG) index, a composite indicator calculated by Ln fasting triglyceride [mg/dl] × Fasting blood glucose [mg/dl]/2, is used to evaluate IR [6]. Unlike traditional IR evaluation methods such as hyperinsulinemic-euglycemic clamp technique and homeostasis model assessment for IR, the TyG index is cheap and easily available [7]. Several studies have shown that it performs consistently with or better than HOMA-IR in evaluating IR [8, 9]. Moreover, TyG index has been found to be associated with CVD [10, 11] and adverse clinical outcomes in patients with conditions such as acute coronary syndrome (ACS), heart failure (HF), and ischemic stroke [12–14]. However, it is still controversy surrounding whether the TyG index, as a marker of IR, can predict the prognosis of CVD patients with different glucose metabolism statuses.

The aim of our study was to discover whether the TyG index has a prognostic value for the risk of all-cause and CVD mortality in in CVD patients with diabetes or pre-diabetes.

## Methods

### Study population and design

**Fig. 1:**
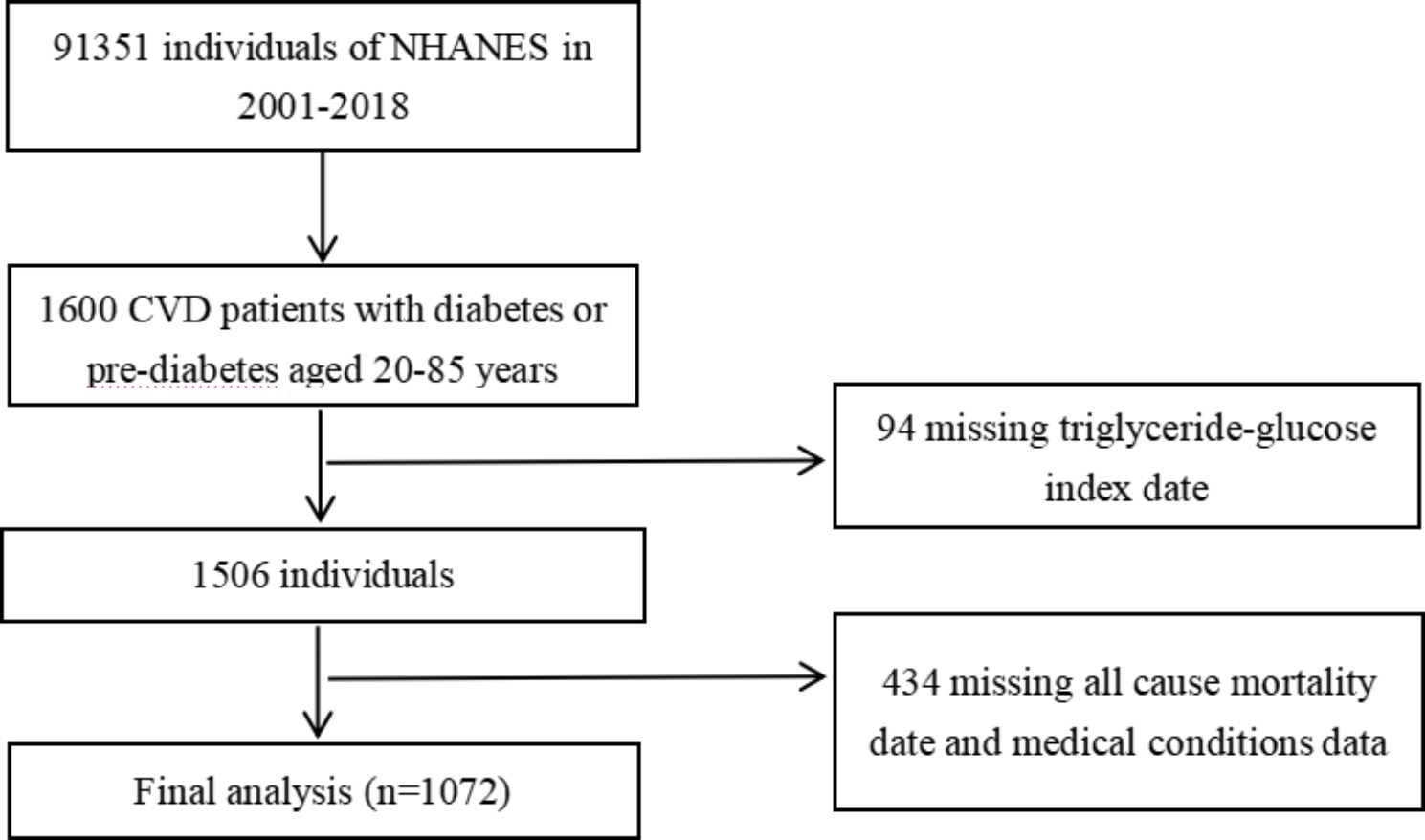
Flow chart of study participants

The National Health and Nutrition Examination Survey (NHANES) is a crucial research program that aims to evaluate the health and nutritional status of both adults and children residing in the United States. The Centers for Disease Control and Prevention (CDC) is responsible for furnishing health statistics for the nation, and the protocols of NHANES have been duly approved by the Research Ethics Review Board of NCHS. To ensure the protection of the participants’ rights, NHANES has obtained informed written consent from all the individuals involved in the study. Moreover, the datasets generated and analyzed in the current study are readily available on the official NHANES website (https://www.cdc.gov/nchs/nhanes/index.html). We downloaded data of NHANES from 2001 to 2018. According to the ADA’s diabetes diagnostic criteria, diabetes is defined by self-reported diagnosis, use of insulin or oral hypoglycemic medication, FBG ≥ 126 mg/dL or HbA1c level ≥ 6.5%. Prediabetes is identified by self-reported prediabetes status or having FBG between 100 mg/dL and 125 mg/dL, or HbA1c between 5.7% and 6.4% [15]. The diagnosis of CVD was established by self-reported physician diagnoses obtained during an individual interview using a standardized medical condition questionnaire. The participants were asked,“Has a doctor or other health expert ever informed you that you have CHF/CHD/angina pectoris/MI/stroke?” A person was regarded as having CVD if he or she replied “yes” to any of the above questions. A total of 1600 adults with CVD and diabetes or pre-diabetes were surveyed (aged 20 to 85 years old). After excluding those who missed triglyceride-glucose index date (n = 94) or missed all cause mortality date and medical conditions data (n = 434) at baseline, 1072 participants were included in the current study (Fig. 1).

### Assessment of covariates

Information on various demographic and health-related factors was gathered, including age, sex, race/ethnicity, education level, family income, smoking status, disease status, and medication use, from NHANES household interviews. Body mass index (BMI) was calculated as weight in kilograms divided by height in meters squared. Race/ethnicity was categorized as White, Black, Mexican, or Other, while education level was classified as less than high school, high school or equivalent, or college or above. Household income and poverty rate are divided into 0–1.0, 1.0–3.0, or > 3.0. Smoking status was recorded as never smoker, former smoker, or current smoker. Drinking status was categorized into heavy drinker (defined as consuming ≥ 3 drinks per day for females, ≥ 4 drinks per day for males, or binge drinking [≥ 4 drinks on the same occasion for females, ≥ 5 drinks on the same occasion for males] on 5 or more days per month), moderate drinker (defined as consuming ≥ 2 drinks per day for females, ≥ 3 drinks per day for males, or binge drinking ≥ 2 days per month), mild drinker (not meeting the above criteria), nondrinker, or a history of daily binge drinking. Clinical indicators such as fasting glucose, HbA1c, triglycerides (TG), total cholesterol (TC), low-density lipoprotein cholesterol (LDL-C), and high-density lipoprotein cholesterol (HDL-C) were measured in the NHANES laboratory.

### Assessment of TyG index

The TyG index was calculated by TyG index = Ln [fasting TG (mg/dL) × fasting glucose (mg/dL)/2]. The measurement of triglycerides and fasting glucose were measured through enzymatic assays on Roche Modular P and Roche Cobas 6000 chemistry analyzers, respectively. The hexokinase-mediated reaction was utilized on Roche/Hitachi Cobas C 501 chemistry analyzers for measuring fasting glucose. The participants were classifed into four groups (Q1, Q2, Q3, Q4) by the quartiles of TyG index, and the Q1 group was used as the reference group.

### Ascertainment of mortality

In order to ascertain the mortality status in the follow-up population, we employed the NHANES public-use linked mortality file as of December 31, 2019. This file was linked with the National Death Index (NDI) by the National Center for Health Statistics (NCHS) via a probability matching algorithm. Moreover, we used the International Statistical Classification of Diseases, 10th Revision (ICD-10) to determine disease-specific deaths, with the NCHS classifying heart diseases (054–064), malignant neoplasms (019–043), and all other causes (010) for our study [16].

### Statistical analysis

The statistical analysis was performed using R software (version 4.2.1; https://www.r-project.org). Sample weights, clustering, and stratification were incorporated in all analyses because of the complex sampling design of the NHANES, as required to analyze the NHANES data [17]. Study participants were classified into four groups according to quartiles (Q1-Q4) of the TyG index. Continuous variables were summarized as mean and standard deviation (SD), while categorical variables were presented as frequency and percentage. The comparison of baseline characteristics across TyG quartile groups was performed using one-way ANOVA for continuous variables and Pearson chi-square test for categorical variables. The incidence rates of all-cause mortality and CVD mortality for each TyG quartile group were computed during the total follow-up period. To evaluate the independent predictive value of the TyG index, we developed multivariate Cox proportional hazards regression models, which included three models to control for confounding factors. Model 1 was unadjusted, Model 2 was adjusted for age, race, and gender, and Model 3 was adjusted for age, gender, race, BMI, tobacco use, alcohol use, education, hypertension, and family income-poverty ratio. Multiple imputation was performed for covariates with missing values. To investigate the relationship between TyG index and mortality, Cox proportional hazards regression models with restricted cubic splines and smooth curve fitting (penalized spline method) were conducted. If the relationship was nonlinear, we estimate the threshold value by trying all possible values and choosing the threshold point with the highest likelihood. And we use two-piecewise Cox proportional risk model on both sides of the inflection point to investigate the association between TyG index and the risk of all-cause mortality and CVD mortality. Stratified analyses were conducted based on gender, age (< 60 years old or ≥ 60 years old), BMI (< 25.00 or ≥ 25.00) and race (White, Black, Mexican, or Other). A p-value of less than 0.05 was considered statistically significant.

## Results

### Baseline characteristics of study participants

Table 1 showed the baseline characteristics of the cohort study participants (n = 1072) stratified by quartiles of the TyG index. The average age of the participants was 66.54 years, and 59.33% of them were male. Average TyG index in the enrolled patients was 9.03 ± 0.03. According to the quartiles of the TyG index, the laboratory characteristics at baseline are shown in Table 2. Participants with a higher TyG index were more likely to be younger, Mexican and obese, compared with participants in the lowest quartile. Additionally, significant differences in Biochemical indicators were observed between the groups, with participants in the highest quartile showing significantly higher levels of HbA1c, LDL-C, TC, TG, GGT, ALT, FINS, and FPG, compared with those in the first quartile.

**Table 1:** Baseline characteristics according to the TyG index quartiles

Characteristics	Quartiles of TyG index					*P* value
	overall	Q1(6.80–8.56)	Q2(8.56–8.96)	Q3(8.96–9.40)	Q4(9.40-12.55)	
**N(%)**	1072	268(25.00)	267(24.90)	269(25.10)	268(25.00)	
**Age, years, mean(SD)**	66.54(0.45)	69.03(0.74)	66.89(0.83)	67.09(0.81)	63.49(0.86)	<0.001
**Gender, n(%)**						
Male	436(40.78)	104(43.45)	108(42.98)	113(40.67)	111(36.38)	0.53
Female	636(59.22)	164(56.55)	159(57.02)	156(59.33)	157(63.62)	
**BMI, kg/m**^**2**^, **n(%)**						<0.001
<24	119(10.93)	48(19.17)	30(11.91)	23( 7.91)	18( 5.95)	
≥24	953(89.07)	220(80.83)	237(88.09)	246(92.09)	250(94.05)	
**Race, n(%)**						0.002
Black	218(11.88)	83(18.95)	53(11.90)	43( 8.84)	39( 8.92)	
Mexican	126( 4.83)	18(3.08)	30(4.66)	33(4.51)	45(6.81)	
White	590(74.22)	132(67.29)	142(71.72)	165(80.88)	151(75.92)	
Other	138( 9.07)	35(10.68)	42(11.72)	28( 5.76)	33( 8.35)	
**Education, n(%)**						0.48
High school grad or equivalent	275(28.83)	62(25.48)	65(28.50)	76(29.15)	72(31.71)	
Less than high school	363(24.97)	79(22.68)	88(23.27)	90(25.53)	106(28.08)	
Some college or above	434(46.20)	127(51.84)	114(48.24)	103(45.31)	90(40.21)	
**Alcohol, n(%)**						0.70
Former	344(30.12)	85(28.23)	86(30.26)	86(29.61)	87(32.12)	
Heavy	105( 9.27)	21( 7.88)	30(11.77)	23( 6.78)	31(10.41)	
Mild	365(39.04)	101(39.71)	88(37.73)	90(41.26)	86(37.57)	
Moderate	85( 7.39)	22(10.43)	20( 6.72)	16( 5.08)	27( 7.85)	
Never	173(14.17)	39(13.75)	43(13.51)	54(17.27)	37(12.05)	
**Smoking status, n(%)**						0.52
Former	434(42.60)	109(42.28)	103(42.82)	111(40.44)	111(44.82)	
Never	446(39.11)	122(44.39)	110(35.84)	110(41.33)	104(35.69)	
Now	192(18.30)	37(13.32)	54(21.34)	48(18.23)	53(19.49)	
**Family poverty income ratio, mean (SD)**	2.54(0.06)	2.50(0.13)	2.46(0.14)	2.60(0.12)	2.61(0.14)	0.86
**Hypertension, n(%)**						0.80
No	181(17.34)	38(14.73)	54(19.07)	41(17.22)	48(17.92)	
Yes	891(82.66)	230(85.27)	213(80.93)	228(82.78)	220(82.08)	
**COPD, n(%)**						0.17
No	941(86.90)	235(87.84)	228(83.20)	231(84.57)	247(92.23)	
Yes	131(13.10)	33(12.16)	39(16.80)	38(15.43)	21( 7.77)	
**DM, n(%)**	744(66.16)	161(55.56)	168(57.13)	189(66.52)	226(84.09)	<0.001
**Pre-DM, n(%)**	328(33.84)	107(44.44)	99(42.87)	80(33.48)	42(15.91)	<0.001

Date are presented as mean (SD) or n (%);

**Table 2:** Baseline levels of laboratory characteristics according to the TyG index quartiles

	Quartiles of TyG index				
	Q1(6.80–8.56)	Q2(8.56–8.96)	Q3(8.97–9.40)	Q4(9.40-12.55)	*P* value
**HbA1C, %, mean (SD)**	6.06(0.07)	6.11(0.10)	6.44(0.08)	7.52(0.15)	<0.001
**eGFR, mL/min/1.73m**^**2**^, **mean (SD)**	69.17(1.69)	74.08(1.74)	72.56(1.59)	75.24(1.70)	0.10
**LDL-cholesterol, mmol/L, mean (SD)**	2.33(0.07)	2.62(0.06)	2.83(0.09)	2.66(0.09)	< 0.001
**HDL-cholesterol, mmol/L, mean (SD)**	1.53(0.04)	1.29(0.03)	1.20(0.02)	1.04(0.02)	< 0.001
**TC, mmol/L, mean (SD)**	4.23(0.08)	4.50(0.07)	4.85(0.09)	5.14(0.10)	< 0.001
**TG, mmol/L, mean (SD)**	0.80(0.02)	1.28(0.02)	1.79(0.03)	3.40(0.16)	< 0.001
**Scr, umol/L, mean (SD)**	107.07(6.57)	92.32(2.65)	93.91(2.47)	94.67(2.78)	0.25
**LDH, IU/L, mean (SD)**	149.69(3.05)	141.46(2.69)	139.17(2.11)	134.52(2.81)	0.01
**Uric acid, umol/L, mean (SD)**	353.11(7.55)	363.86(6.64)	375.61(7.85)	371.84(8.92)	0.22
**Albumin, g/L, mean (SD)**	40.98(0.25)	41.50(0.23)	41.19(0.24)	41.18(0.23)	0.43
**FINS, pmol/L, mean (SD)**	74.09(4.40)	106.28(9.51)	111.25(7.45)	143.24(9.77)	< 0.001
**FPG, mmol/L, mean (SD)**	6.10(0.07)	6.58(0.12)	7.20(0.12)	9.75(0.27)	< 0.001
**Serum potassium, mmol/L, mean (SD)**	4.15(0.04)	4.14(0.03)	4.17(0.03)	4.18(0.03)	0.67
**Serum iron, umol/L, mean (SD)**	15.09(0.51)	15.69(0.47)	14.95(0.36)	15.19(0.41)	0.61
**Serum sodium, mmol/L, mean (SD)**	139.71(0.23)	139.46(0.21)	139.32(0.21)	138.47(0.28)	0.01
**BUN, mmol/L, mean (SD)**	6.67(0.23)	6.18(0.20)	6.20(0.21)	6.69(0.24)	0.12
**AST (IU/L)**	27.50(2.36)	26.02(0.69)	26.36(1.16)	25.58(0.58)	0.79
**ALT (IU/L)**	22.28(1.04)	24.62(0.96)	25.75(1.28)	27.08(0.80)	0.004
**TBil, umol/L, mean (SD)**	13.20(0.45)	12.96(0.49)	12.63(0.30)	12.11(0.39)	0.31
**GGT (IU/L)**	29.63(2.08)	39.31(4.13)	39.54(5.50)	43.76(3.35)	0.001

Date are presented as mean (SD) or n (%);

### Relationships of TyG index concentration with mortality

Table 3 presents the occurrence of 461 all-cause deaths and 154 CVD-related deaths during the follow-up period. We constructed three Cox regression models to investigate the independent association between TyG index levels and mortality risk. After adjusting for age, gender, race, BMI, tobacco use, alcohol use, education, hypertension, and family income-poverty ratio in Model 3, the multivariate-adjusted hazard ratios (HRs) and 95% confidence intervals (CIs) from lowest to highest TyG index quartile (6.80–8.56, 8.56–8.96, 8.96–9.40, and 9.40-12.55) were 1.00 (reference), 0.65 (0.45, 0.93), 0.84 (0.62, 1.14), and 1.3 (0.73, 1.26), respectively, for all-cause mortality (P = 0.623); 1.00 (reference), 0.49 (0.25, 0.97), 1.10 (0.63, 1.90), and 1.30 (0.73, 2.33), respectively, for CVD mortality (P = 0.068).

**Table 3:** HRs (95% CIs) for mortality according to the TyG index quartiles

	Quartiles of TyG index				
	Q1(6.80–8.56)	Q2(8.56–8.96)	Q3(8.96–9.40)	Q4(9.40-12.55)	*P* trend
**All-cause mortality**					
Number of deaths	119	104	122	116	
Model 1	1	0.61(0.42,0.89) 0.01	0.77(0.57,1.03) 0.08	0.76(0.57,1.01) 0.06	0.29
h(95%CI) P-value					
Model 2	1	0.66(0.46,0.95) 0.02	0.86(0.64,1.17) 0.34	1.00(0.75,1.32) 0.99	0.46
h(95%CI) P-value					
Model 3	1	0.63(0.44,0.91) 0.01	0.84(0.62,1.13) 0.25	1.01(0.77,1.32) 0.94	0.36
h(95%CI) P-value					
**CVD mortality**					
Number of deaths(%)	40	24	47	43	
Model 1	1	0.35(0.17,0.73) 0.01	0.84(0.50,1.40) 0.05	0.88(0.51,1.51) 0.64	0.49
h(95% CI) P-value					
Model 2	1	0.37(0.18,0.75) 0.01	0.96(0.57,1.62) 0.89	1.12(0.65,1.96) 0.68	0.14
h(95% CI) P-value					
Model 3	1	0.37(0.18,0.78) 0.01	1.13(0.65,1.96) 0.67	1.28(0.71,2.30) 0.41	0.04
h(95% CI) P-value					

Model 1: Non-adjusted

Model 2: Adjusted for age, race and gender

Model 3: Adjusted for age, gender, race, BMI, tobacco use, alcohol use, education, hypertension, COPD, family income-poverty ratio. HR: Hazard ratio; CI: Confidence interval

### The detection of nonlinear relationships

**Fig. 2:**
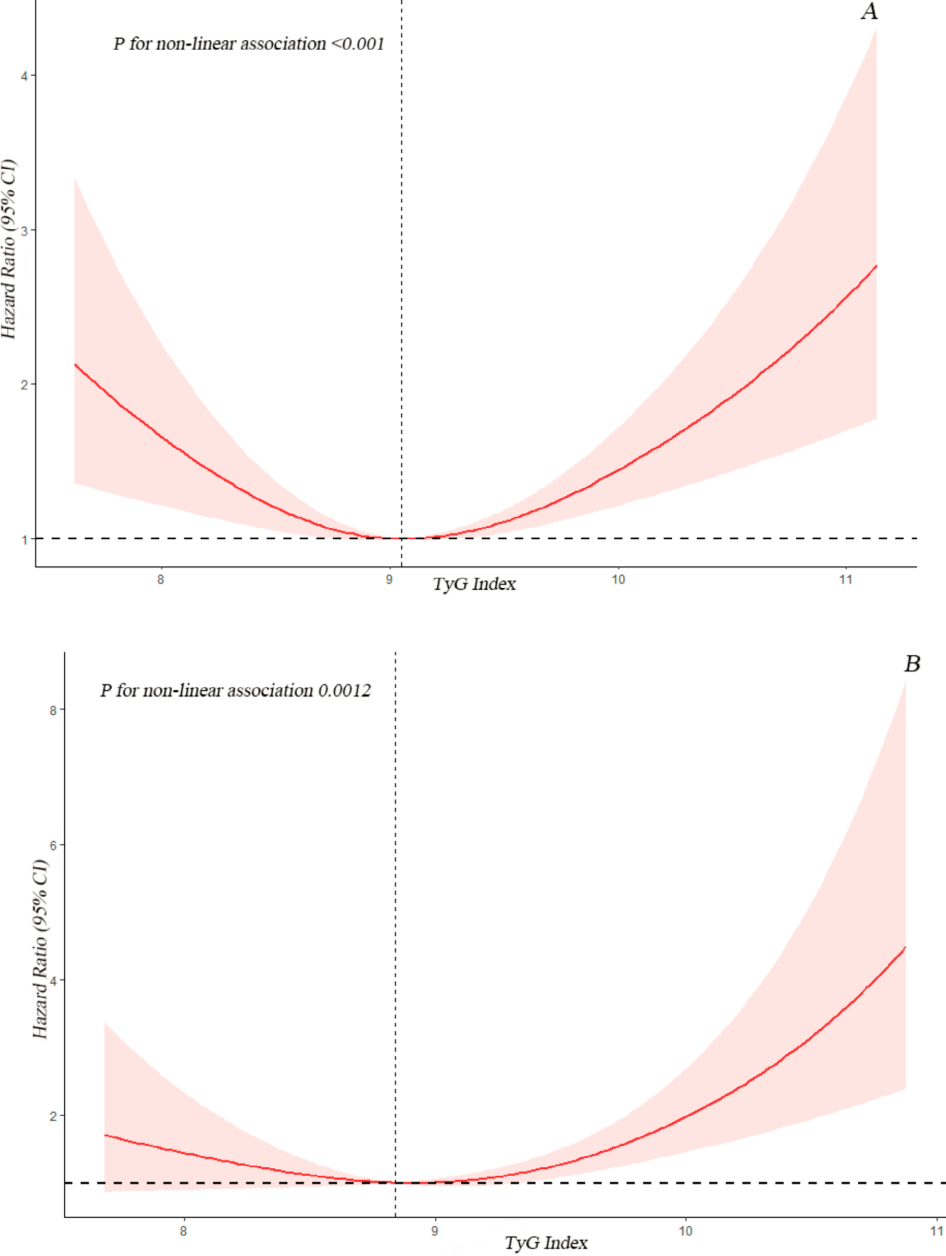
Association between TyG index and all-cause (**A**) and CVD mortality (**B**) in CVD patients with diabetes or pre-diabetes. Each hazard ratio was computed with a TyG index level of A 9.05 and B 8.84 as the reference. Adjusted for age, gender, race, BMI, tobacco use, alcohol use, education, hypertension, family income-poverty ratio. The solid line and red area represent the estimated values and their corresponding 95% CIs, respectively (TyG index: triglyceride-glucose index; CVD: cardiovascular disease)

**Fig. 3:**
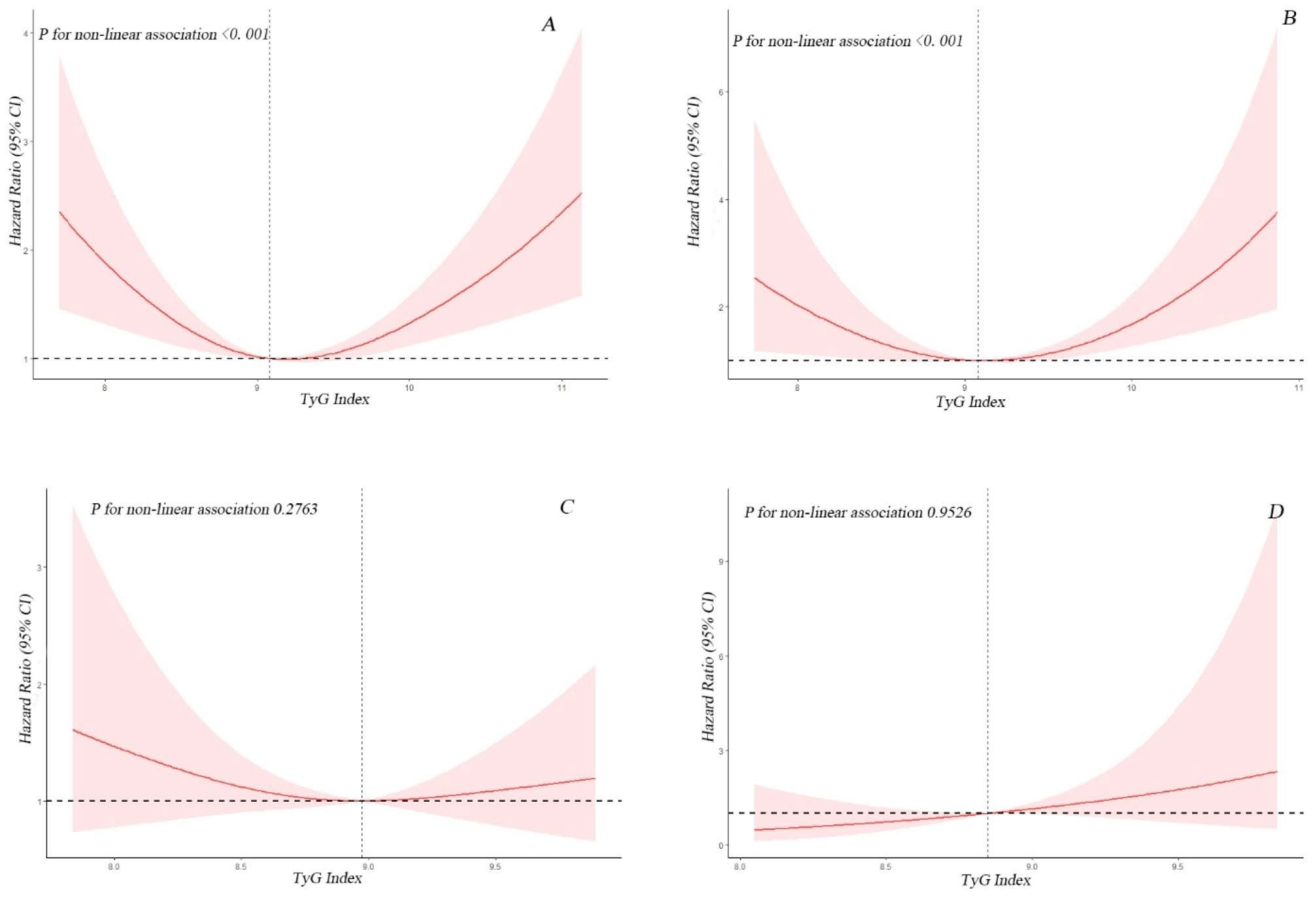
Association between TyG index and all-cause (**A**) and CVD mortality (**B**) in CVD patients with diabetes. Each hazard ratio was computed with a TyG index level of A 9.08 and B 9.08 as the reference. Association between TyG index and all-cause (**C**) and CVD mortality (**D**) in CVD patients with pre-diabetes. Each hazard ratio was computed with a TyG index level of A 8.97 and B 8.85 as the reference. Adjusted for age, gender, race, BMI, tobacco use, alcohol use, education, hypertension, family income-poverty ratio. The solid line and red area represent the estimated values and their corresponding 95% CIs, respectively (TyG index: triglyceride-glucose index; CVD: cardiovascular disease)

Due to previous multivariate analysis indicated a non-linear relationship between baseline TyG index and the risk of all-cause and CVD mortality, we employed a Cox proportional hazards regression models with restricted cubic splines and smooth curve fitting (penalized spline method) to further investigate the correlation. Interestingly, the adjusted smoothed plots displayed U-shaped associations between TyG index and all-cause (Fig. 2A) and CVD mortality (Fig. 2B). We fitted the association between baseline TyG index and mortality using the standard Cox proportional hazards regression models and the the two-piecewise Cox proportional hazards regression models. Based on the two-piecewise Cox proportional hazards regression models, we identified the inflection points for all-cause and CVD mortality as 9.05 and 8.84, respectively (both P values for log-likelihood ratio < 0.05) (Table 4). After adjusting for age, gender, race, BMI, tobacco use, alcohol use, education, hypertension, and family income-poverty ratio, the risk of all-cause and CVD mortality decreased by approximately 53% (HR 0.47, 95% CI = 0.27–0.81) and 75% (HR 0.25, 95% CI = 0.07–0.89), respectively, with each unit increase in the TyG index up to the inflection points. Furthermore, the risk of all-cause and CVD mortality decreased to the lowest level as the baseline TyG index increased up to the threshold values (Table 4; Fig. 2). Conversely, the baseline TyG index was significantly and positively associated with the risk of all-cause and CVD mortality when it exceeded 9.05 (HR 1.42, 95% CI = 1.02–1.99) and 8.84 (HR 1.77, 95% CI = 1.08–2.91), respectively. We studied the population of diabetes and pre-diabetes separately. The results showed that there was still a U-shaped relationship between the TyG index and the all-cause mortality and CVD mortality in CVD patients with diabetes (both P for non-linear < 0.01). The TyG index and the all-cause mortality in CVD patients with pre-diabetes approximate a U-shaped relationship (P for non-linear = 0.28), while there was an approximate linear relationship with CVD mortality (P for non-linear = 0.95) Fig. 3.

**Table 4:** Threshold effect analysis of TyG index on all-cause and CVD mortality in CVD patients with diabetes or pre-diabetes

	Adjusted HR (95% CI), *P*-value
**All-cause mortality**	
Total	1.12(0.92,1.35) 0.25
Fitting by two-piecewise Cox proportional risk model	
Inflection point	9.05
TyG index < 9.05	0.47(0.27,0.81) 0.01
TyG index ≥ 9.05	1.42(1.02,1.99) 0.04
*P* for Log-likelihood ratio	< 0.001
**CVD mortality**	
Total	1.53(1.08,2.18) 0.02
Fitting by two-piecewise Cox proportional risk model	
Inflection point	8.84
TyG index < 8.84	0.25(0.07,0.89) 0.03
TyG index ≥ 8.84	1.77(1.08,2.91) 0.02
*P* for Log-likelihood ratio	< 0.001

Cox proportional hazards models were used to estimate HR and 95% CI. Adjusted for age, gender, race, BMI, tobacco use, alcohol use, education, hypertension, COPD, family income-poverty ratio. HR: Hazard ratio; CI: Confidence interval

### Stratified analyses

The survival advantage of a higher TyG index (≥ 9.05 for all-cause mortality and ≥ 8.84 for CVD mortality) compared to a lower TyG index (< 9.05 for all-cause mortality and <8.84 for CVD mortality9.05) among CVD patients with diabetes or pre-diabetes was consistent across various subgroups based on age, gender, race, and BMI, as depicted in Tables 5 and 6. There was no significant interaction between the baseline TyG index and stratified variables.

**Table 5:** Stratified analyses of the associations between TyG and All mortality

	All-cause mortality		
	HR(95% CI) P-value		
TyG index	< 9.05	>=9.05	*p* interaction
**Overall**	1	1.18(0.95,1.48) 0.13	
**gender**			0.53
Female	1	1.26(0.83,1.91) 0.28	
Male	1	1.89(1.16,3.07) 0.01	
**Age, years**			0.12
< 60	1	1.29(0.69,2.44) 0.42	
>=60	1	0.98(0.78,1.24) 0.86	
**BMI, kg/m2, n(%)**			0.99
<24	1	0.94(0.56,1.57) 0.82	
>=24	1	1.20(0.95,1.51) 0.13	
**Race**			0.78
Black	1	1.51(0.98,2.31) 0.06	
Mexican	1	0.93(0.43,2.00) 0.85	
Other	1	0.90(0.34, 2.38) 0.83	
White	1	1.16(0.89,1.50) 0.27	
**DM**			0.59
T2DM	1	1.15(0.90,1.46) 0.27	
Pre-dm	1	1.04(0.64,1.64) 0.85	

**Table 6:** Stratified analyses of the associations between TyG and CVD mortality

	CVD mortality		
	HR(95% CI) P-value		
TyG index	< 8.84	>=8.84	*p* interaction
**Overall**	1	1.70(1.16,2.50) 0.01	
**gender**			0.61
Female	1	1.55(0.77, 3.12) 0.22	
Male	1	1.89(1.16,3.07) 0.01	
**Age, years**			0.39
< 60	1	2.12(0.45,9.98) 0.34	
>=60	1	1.18(0.76,1.81) 0.46	
**BMI, kg/m2, n(%)**			0.14
<24	1	0.56(0.17,1.85) 0.34	
>=24	1	2.10(1.30,3.38) 0.002	
**Race**			0.74
Black	1	1.91(0.93, 3.93) 0.08	
Mexican	1	2.93(0.70,12.23) 0.14	
Other	1	9.05(0.69, 118.85) 0.09	
White	1	1.59(0.97,2.91) 0.06	
**DM**			0.31
T2DM	1	1.33(0.87,2.01) 0.18	
Pre-dm	1	2.85(1.28,6.33) 0.01	

## Discussion

To our knowledge, this is the first study to revealed a U-shaped association between the baseline TyG index with all-cause and CVD mortality among CVD patients with diabetes or pre-diabetes. We revealed a turning point (9.05 in all-cause mortality and 8.84 in CVD mortality) using threshold effect analysis. Our study shows that the TyG index is a strong predictor of all-cause and cardiovascular mortality among CVD patients with diabetes or pre-diabetes.

Previous clinical studies have also explored the association between the TyG index with CVD morbidity and mortality in various patient groups and the general population. A study conducted by Zhai [18] demonstrated that the TyG index was a strong predictor of in-hospital mortality in critically ill patients with CVD. And for patients with stable cardiovascular disease, a positive association was observed between the TyG index and adverse cardiovascular outcomes [19]. Additionally, in 2010, Guerrero et al. [20] demonstrated the potential of the TyG index as an estimate of IR, exhibiting a high degree of correlation with the glucose clamp technique and remarkable sensitivity and specificity. As a result, the TyG index offers distinct advantages over other indices in evaluating insulin resistance [21].

While the exact biological mechanisms underlying the correlation between the TyG index and mortality remain unclear, possible key pathways may be related to IR, a state of reduced sensitivity and responsiveness to insulin action. IR as a risk factor for CVD, can leads to the development of CVD in both the general population and diabetes patients and predicts cardiovascular prognosis in patients with CVD [22]. patients with IR have an increased risk of developing metabolic disorders such as hyperglycemia, dyslipidemia, and hypertension, all of which are strongly linked to poor CVD outcomes [23]. The chronic hyperglycemia and dyslipidemia that arise from IR can trigger oxidative stress, exacerbate inflammatory responses, promote foam cell formation, impair endothelial function, and encourage smooth muscle cell proliferation [24]. Furthermore, persistent IR can increases sympathetic nervous system activity, renal sodium retention and elevated blood pressure, which can increases cardiac load, leads to vascular and renal damage [25]. These pathological changes can contribute to the development and progression of coronary heart disease (CHD) and lead to a poor prognosis. Our study revealed a positive correlation between the TyG index and BMI, FBG, HbA1c, TG, total cholesterol, and LDL, as well as a negative correlation with HDL. These results suggest that the relationship between the TyG index and poor outcomes may be attributed to the presence of traditional CVD risk factors, which has been reported previously [26]. Taken together, our findings support the utility of the TyG index as a reliable and accurate indicator of IR for risk stratification in the real world.

Interestingly, lower levels of baseline TyG index (TyG index < 9.05 for all-cause mortality and < 8.84 for CVD mortality) significantly altered the correlation between TyG index and risk of all-cause and CVD mortality. After adjusting for confounding factors, each unit increase in baseline TyG index decreases the risk of all-cause and CVD mortality by almost 53% and 75%, respectively, in participants whose baseline TyG index is below the threshold. There were evidences that extremely low levels of TG or FPG are associated with adverse effects on health and may contribute to the development of diseases [27]. Hypoglycaemia has been shown to increase levels of counter-regulatory hormones such as adrenaline, leading to vasoconstriction and platelet aggregation, causing an increase in cardiovascular or cerebrovascular stroke events [28]. Moreover, a prospective cohort study revealed that low serum TG levels were linked to an elevated risk of hemorrhagic stroke in women [29]. Similarly, low TG levels were identified as a predictor of cardiac death in patients with heart failure [30]. Additionally, Jin Shang’s research demonstrated a non-linear association between the TyG index and the risk of developing newly diagnosed diabetic nephropathy, indicating that both extremely low and high levels of TyG increased the incidence of diabetic nephropathy [31]. Therefore, it is important to maintain an appropriate level of TyG index since both excessively high and low levels may have negative health consequences. We further studied the population with diabetes and pre-diabetes separately, and the results showed that there was still a U-shaped relationship between the TyG index and the all-cause mortality and CVD mortality of the diabetes population, and the results were statistically significant. The TyG index and the all-cause mortality rate of patients with pre-diabetes are similar to U-shaped relationship, but the results are not statistically significant. The above difference may be that the number of pre-diabetes patients with cardiovascular disease included in this study is small, which requires a larger sample study in the future. There are limited data on the associations between the TyG index and all-cause and CVD mortality in CVD patients with diabetes or pre-diabetes. Although this is the first study to report a U-shaped association between the baseline TyG index and CVD and all-cause mortality in CVD patients with diabetes or pre-diabetes in a US cohort. There are several limitations to this study. As an observational study conducted in a single center, it cannot establish causality definitively. Although we have attempted to control for confounding variables through multivariate adjustment and subgroup analyses, there may be residual confounding factors that could impact the prognosis. Furthermore, this analysis only examines the prognostic value of the baseline TyG index, and it is unclear whether changes in the TyG index during follow-up also predict mortality, which requires further investigation.

## Conclusion

The results of our study indicate that the TyG index is a valuable tool for predicting the risk of all-cause and CVD mortality in CVD patients with diabetes or pre-diabetes, and that the association between the TyG index and mortality is non-linear. Therefore, measuring the TyG index may be beneficial in assessing the risk and predicting the prognosis of this patient population. Future studies should explore whether interventions targeting the TyG index could lead to improved clinical outcomes in these patients.

## Data Availability

The datasets that were used and evaluated in this study can be obtained from the corresponding author upon making a reasonable request.

## Abbreviations

ACS: Acute coronary syndrome
ALT: Alanine aminotransferase
BMI: Body mass index
CDC: The Centers for Disease Control and Prevention
CHD: Coronary heart disease
CHF: Congestive heart failure
CI: Confidence interval
CV: Cardiovascular
CVD: Cardiovascular disease
DBP: Diastolic blood pressure
FBG: Fasting blood glucose
FINS: Fasting insulin
GGT: γ-glutamyl transpeptadase
HbA1c: Glycosylated hemoglobin A1c
HDL-C: High-density lipoprotein cholesterol
HF: Heart failure
HOMA-IR: Homeostasis model assessment of insulin resistance
HR: Hazard ratio
IR: Insulin resistance
LDL-C: Low-density lipoprotein cholesterol
MI: Myocardial Infarction
NCHS: National Center for Health Statistics
NDI: National Death Index
NHANES: National Health and Nutrition Examination Survey
SBP: Systolic blood pressure
SD: Standard deviation
T2DM: Type 2 diabetes mellitus
TC: Total cholesterol
TG: Triglyceride
TyG: Triglyceride-glucose index
